# DNA cleavage by CgII and NgoAVII requires interaction between N- and R-proteins and extensive nucleotide hydrolysis

**DOI:** 10.1093/nar/gkv049

**Published:** 2015-03-13

**Authors:** M. Zaremba, P. Toliusis, R. Grigaitis, E. Manakova, A. Silanskas, G. Tamulaitiene, M.D. Szczelkun, V. Siksnys

Nucleic Acids Res. 2014 Dec 16;42(22):13887–96. doi: 10.1093/nar/gku1236.

## Crystal structure of the R-protein of the multisubunit ATP-dependent restriction endonuclease NgoAVII

TamulaitieneG.SilanskasA.GrazulisS.ZarembaM.SiksnysV.

Nucleic Acids Res. 2014 Dec 16;42(22):14022–30. doi: 10.1093/nar/gku1237.

Throughout these articles, we have used the prefix ‘N’ (as in N.CglI and N.NgoAVII) to describe NTPases associated with the cognate restriction systems. However, this prefix is also used for nicking restriction enzymes as described in (1). To avoid confusion, we therefore wish to alter the nomenclature used in this article and in the future, by replacing ‘N’ with the prefix ‘H’, to indicate a subunit that contains a domain with amino acid motifs characteristic of a helicase, with activities not limited to unwinding. The list of affected enzymes is provided below (Table 1). The results and conclusion of these articles are not affected by this correction.

**Table 1.** Changes in nomenclature.

Old name*	New name*
**N.**CglI	**H.**CglI
**N.**NgoAVII	**H.**NgoAVII

*Changes are shown in bold.
